# Mutation of NIMA-related kinase 1 (NEK1) leads to chromosome instability

**DOI:** 10.1186/1476-4598-10-5

**Published:** 2011-01-10

**Authors:** Yumay Chen, Chi-Fen Chen, Huai-Chin Chiang, Michelle Pena, Rosaria Polci, Randy L Wei, Robert A Edwards, Donna E Hansel, Phang-Lang Chen, Daniel J Riley

**Affiliations:** 1 Department of Medicine, Division of Endocrinology, University of California at Irvine, 1130 Gross Hall, Irvine, CA 92697, USA; 2 Department of Biological Chemistry, University of California at Irvine, Medical Science 1, Irvine, CA, 92697, USA; 3 Department of Medicine, Division of Nephrology, The University of Texas Health Science Center at San Antonio, 7703 Floyd Curl Drive, San Antonio, TX 78229, USA; 4 Current Address: Department of Nephrology and Dialysis, Belcolle Hospital, Viterbo, Italy; 5 Department of Pathology and Lab Medicine, University of California at Irvine, Medical Science 1, Irvine, CA 92697, USA; 6 Department of Anatomic Pathology, Cleveland Clinic, 9500 Euclid Ave. Cleveland, OH 44195, USA; 7 Transplant Center, The University of Texas Health Science Center at San Antonio, 7703 Floyd Curl Drive, San Antonio, TX 78229, USA

## Abstract

**Background:**

NEK1, the first mammalian ortholog of the fungal protein kinase never-in-mitosis A (NIMA), is involved early in the DNA damage sensing/repair pathway. A defect in DNA repair in NEK1-deficient cells is suggested by persistence of DNA double strand breaks after low dose ionizing radiation (IR). NEK1-deficient cells also fail to activate the checkpoint kinases CHK1 and CHK2, and fail to arrest properly at G1/S or G2/M-phase checkpoints after DNA damage.

**Results:**

We show here that NEK1-deficient cells suffer major errors in mitotic chromosome segregation and cytokinesis, and become aneuploid. These NEK1-deficient cells transform, acquire the ability to grow in anchorage-independent conditions, and form tumors when injected into syngeneic mice. Genomic instability is also manifest in *NEK1 *+/- mice, which late in life develop lymphomas with a much higher incidence than wild type littermates.

**Conclusion:**

NEK1 is required for the maintenance of genome stability by acting at multiple junctures, including control of chromosome stability.

## Background

Cancers frequently develop abnormal numbers of chromosomes and contain chromosomal rearrangements. This genomic instability generates daughter cells that die because of insufficient complements of chromosomes, as well as polyploid cells that acquire mutations favorable for uncontrolled proliferation. Genomic instability is less frequently observed in non-cancerous cells, which have competent surveillance mechanisms to monitor errors in DNA replication and chromosome segregation during mitosis, as well as the machinery to repair such damage. Dysregulation of these two important mechanisms leads to genomic instability, and ultimately to increased mutation rates and acquisition of the multiple mutations that lead to cancer.

Mitotic protein kinases, such as never-in-mitosis A (NIMA) in fungi and NIMA-related kinases (Neks or Nrks) [1] in mammals, have been implicated in guarding the integrity of the genome. NIMA functions as a protein kinase, regulates G2-M phase progression, increases expression in response to DNA damage, and serves to ensure proper mitotic spindle organization and formation of the nuclear envelope [2-4]. There are 11 known mammalian NEKs. NEK2 is the one best characterized to date. It has been shown to have a role in controlling orderly mitosis and in preventing chromosomal instability [1,5,6]. NEK6 and NEK7 have been implicated in regulating mitotic progression [7,8]. Nek8, like NEK1, has been linked genetically with a form of polycystic kidney disease; it localizes to the primary cilium of each cell where it functions to anchor mitotic centrosomes [9-12]. NEK11 has been linked to the CDC25A degradation in response to DNA damage and is a substrate of CHK1 [13]. Thus, like their lower eukaryotic orthologs, the NEK family of kinases has many members. Each seems to have its unique cellular function, a function required for orderly progression through the cell division cycle.

Recently, we uncovered a role for NEK1 in DNA damage responses [14,15]. NEK1 is a dual serine-threonine and tyrosine kinase [16] and its kinase activity and expression are quickly upregulated in cells treated with IR. Within minutes after exposure to IR or other genotoxic agents, a portion of NEK1 redistributes from the cytoplasm into the nucleus, where it forms discrete nuclear foci at sites of DNA damage. NEK1 colocalizes with γ-H2AX and MDC1/NFBD1, which are among the first responders to IR-induced double strand breaks (DSBs). The importance of NEK1 in the DNA damage signaling pathway was revealed by analyzing cells lacking functional NEK1. These cells fail to activate downstream checkpoint proteins, such as CHK1/CHK2, and fail to arrest at S or G2/M phase to allow for efficient DNA repair [14,15]. Consequently, *NEK1*-deficient cells develop many more chromosome breaks than wild type cells [14,15].

Because *NEK1 *mRNA is abundantly expressed in mouse gonads and neurons [16], early reports suggested that NEK1 protein functions in a direct and unique way in meiosis or in regulating the cell division cycle [17,18]. Whether NEK1 plays a role in regulating chromosomal stability is still unknown at this time. Neither is it known whether NEK1 functions as a tumor suppressor like many checkpoint/mitotic kinases (CHK1, Mps1, and BubR1). In this report, we demonstrate that NEK1 is important for genomic and chromosome stability. Cells defective in NEK1 suffer from disordered mitosis, become aneuploid after multiple cell division cycles, and acquire transforming activity. NEK1 also seems to function as a tumor suppressor, since mice heterozygous for a *NEK1/kat2J *mutation develop tumors, specifically lymphomas, with a much higher incidence compared to their wild type littermates.

## Methods

### Cell culture

Primary renal tubular epithelial cells (RTEs) and tail fibroblasts were obtained from *NEK1-*mutated kat2J mice and their wild type littermates as previously described [15], and cultured in the Ham's F-12/DMEM.

### Antibodies

Anti-α-tubulin mAb DM1A and rabbit anti-mouse *CD3 *were purchased from Sigma-Aldrich (St. Louis, MO, USA), rabbit anti-*CD45R *antibodies from Abcam, Inc. (Cambridge, MA, USA), rabbit anti-p19ARF antibodies from Genetex (Irvine, CA, USA), fluorochrome-conjugated secondary antibodies (Alexa-Fluoro 488 for green, Alexa-Fluoro 594 for red) from Molecular Probes, Inc. (Eugene, OR, USA), and horseradish peroxidase-based secondary antibodies from Vector Technologies (Burlingame, CA, USA).

### Immunocytochemistry

Cells grown on coverslips to 60% confluence were fixed in 4% formaldehyde with 0.1% Triton X-100. Fixed cells were permeabilized with 0.05% saponin and blocked with 10% normal goat serum. Primary antibodies were used at a dilution of 1:100 to 1:1,000 (3 to 0.3 μg/mL) in 10% goat serum. Secondary antibodies, including anti-rabbit or anti-mouse IgG-Alexa 594 (red) and anti-rabbit or anti-mouse IgG-Alexa 488 (green; Molecular Probes, Eugene, OR, USA) were used at a dilution of 1:3,000. Cells were mounted in Permafluor (Lipshaw-Immunon, Pittsburgh, PA, USA). Images were captured with a Ziess AxioPlan2 fluorescence microscope and digitally merged where appropriate.

### Chromosome spreads

Mouse RTEs in logarithmic growth phase were treated with colchicine (1 μg/ml, from Sigma) for 30 min at 37°C. All cells, including those in the supernatants, were then collected by trypsinization and swollen in 75 mM KCl for 15 min at 37°C. Disbursed cells were then fixed with freshly prepared methanol: acetic acid (3:1). Free chromosomes were dropped onto slides and stained with Giemsa.

### Flourescence-activated cell sorting

Monolayers of the same primary RTEs were trypsinized, washed with Ham's F12/DMEM containing serum to inactivate the trypsin, and then washed with PBS. Spleen and lymohoma tissues were disrupted in PBS using a rubber policeman. The tissue homogenates were passed through 70-μm filter to obtain single cell suspensions. Cells were well suspended in 1 ml of PBS before fixation with ethanol to a final concentration of 70%. The fixed cells were washed again with PBS, treated with RNase, and labeled with propidium iodide (1 μg/ml) before sorting by a Becton Dickinson instrument.

### Soft agar colony formation

Soft agar colony formation assays were performed as previously described [15]. Equal numbers of cells (1 × 10^5 ^or 2 × 10^4^) from each of the indicated cell types at different passage were seeded in 0.367% agar. After 21 days of incubation at 37°C, colonies containing at least 50 cells were counted and representative colonies were photographed.

### In Vivo Tumor Growth

Monolayers of the RTEs at passage 7 were trypsinized and resuspended in 1 ml of PBS at a density of 2.5 × 10^7^/ml. 5 × 10^6 ^cells were injected subcutaneously into the flanks of *NEK1 *+/- kat 2J mice. Tumor formation was observed at intervals starting at 7 days later and tumors were harvested at day 10 or 21 for histological analysis.

### *NEK1*-mutated kat2J mice and genotyping

C57BL/6J-*NEK1kat2J +/- *founder mice were obtained from The Jackson Laboratory (Bar Harbor, ME, USA). Genomic DNA was extracted from 1 × 10^4 ^cultured cells, tail fragments tissue, or blood, according to a protocol available at http://www.jax.org/imr/tail_nonorg.html. Details of the genotyping for the kat2J mutation, a single guanine insertion at nucleotide +996 that results in a truncated, unstable protein missing the entire kinase domain [9], and the single-strand conformational polymorphism (SSCP) analysis have already been described in detail [14,15]. We have since modified the published protocol only to eliminate the need to use radioactive isotope in the detection of bands on polyacrylamide gels. Gels were fixed with 10% methanol/10% glacial acetic acid, stained with 4× gel red dye, and visualized and photographed with a UVP image system.

### Generation of inducible knockdown and retrovirus expression constructs

An inducible shRNAi construct to knock down NEK1 expression was designed and made as previously described [19]. Briefly, Nek1 shRNA expression cassette was created in pTER (pTER-NEK1i). Four such cassettes from pTER-NEKi were inserted in tandem into pPUR (pPUR-4xNEK1i). U2-OS cells with inducible NEK1 shRNA [14] were established by Lipofectamine 2000-mediated transfection of pPUR-4xNEK1i and a TetR-expressing construct, pCDNA6TR, followed by selection with 5.0 μg/ml blasticidin and 5.0 μg/ml puromycin. N27 was isolated as an inducible NEK1 knockdown clone initially, but later became a line with constitutive knockdown. Passage number 1 was defined as the cells when they were first established to knock down NEK1 expression stably. For the retrovirus expressing wild-type NEK1, GFP-tagged NEK1 was subcloned into retroviral vector pQUXIP, with a modification to replace the CMV-IE promoter with a UBC promoter. Retrovirus was produced by co-transfection of pQUXIP-GFP-Nek1 and VSV-G into 293-GP2 cells.

### Histology and immunohistochemistry

Different tissues were harvested immediately after mice were euthanized, and fixed overnight in 10% neutral buffered formalin at 4°C. After progressive dehydration and embedding in paraffin, 3-μm sections were stained with Meyer's hematoxylin and eosin reagents. For immunohistochemical staining, 4-μm tissue sections on slides were deparaffinized with Histoclear (National Diagnostics, Atlanta, GA, USA) and rehydrated with graded ethanol. Primary antibodies including anti-CD3 and anti-CD45R were used to stain for markers of T or B lymphocytes. Biotinylated secondary, anti-mouse and anti-rabbit IgG antibodies and immuno-peroxidase-based ABC development kits were purchased from Vector Laboratories (Burlingame, CA, USA). Immunoperoxidase-stained sections were then counterstained with methyl green to identify nuclei.

## Results

### Chromosome instability in NEK1-deficient cells

**Figure 1:**
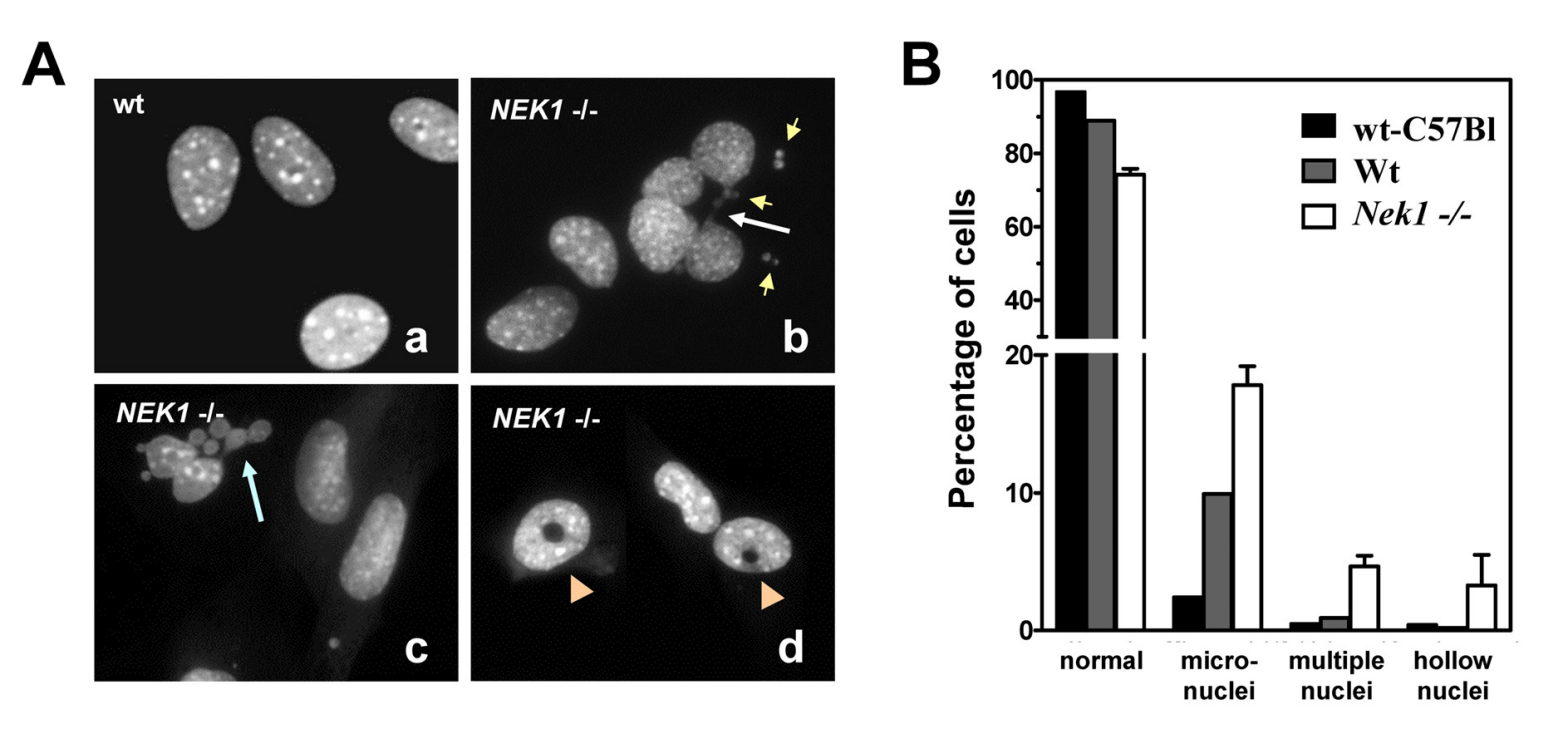
**Genomic instability in NEK1 deficient cells**. A. Renal tubular epithelial cells from wild type and NEK1/kat2J -/- mice were fixed with 4% paraformaldehyde during exponential phase growth and stained with DAPI for nuclear morphology Almost all wild type cells had normal nuclear morphology (panel a). *NEK1 *-/- cells often had micronuclei (panel b, small arrows), multiple nuclei (panel c, arrows), or hollow nuclei (panel d, arrow heads). Chromosome bridges were also frequently observed (panel b, large arrow). B. Each histogram represents percentages (means ± standard errors) of each type of abnormal nuclei from 2 distinct cell lines at passage 4, and totals of 1000 cells scored per cell line.

Cells with defective spindle checkpoints or defective DNA repair mechanisms fail to arrest cell cycle progression in time to allow proper DNA repair. Thus they fail to allow the damage to be repaired before dividing. Consequently, unrepaired DNA results in micronucleated cells, which are markers for genomic instability [19,20]. To investigate NEK1's role in checkpoint activation further, we performed detailed analysis of cells without functional NEK1. Primary RTE cells established from kat2J mice [9], in which both *NEK1 *alleles are mutated (*NEK1 *-/-), and from wild type littermates (*NEK1 *+/+) were examined after staining with DAPI to reveal their nuclear morphology (Figure 1). Evidence of DNA damage owing to cell cycle checkpoint defects was observed in the interphase nuclei of many *NEK1 *-/- cells. Multiple nuclei, micronuclei, bridging chromosomes, and unique hollow nuclei were prominent (Figure 1A, *panels b-d*). Morphologically normal nuclei were observed in the vast majority of cells from wild type littermate mice or from similarly aged C57Bl/6J mice, the strain from which kat2J mice were generated (Figure 1A, *panel a*). Cells established from C57Bl/6 mice were used to make sure that observed abnormalities did not stem only from inbreeding in the NEK1/kat2J strain. *NEK1 *-/- cell lines, RTEs and tail fibroblasts, were established from at least 8 different mice, and similar abnormalities were observed in each of them.

### *NEK1 *mutation results in disordered mitosis

**Figure 2:**
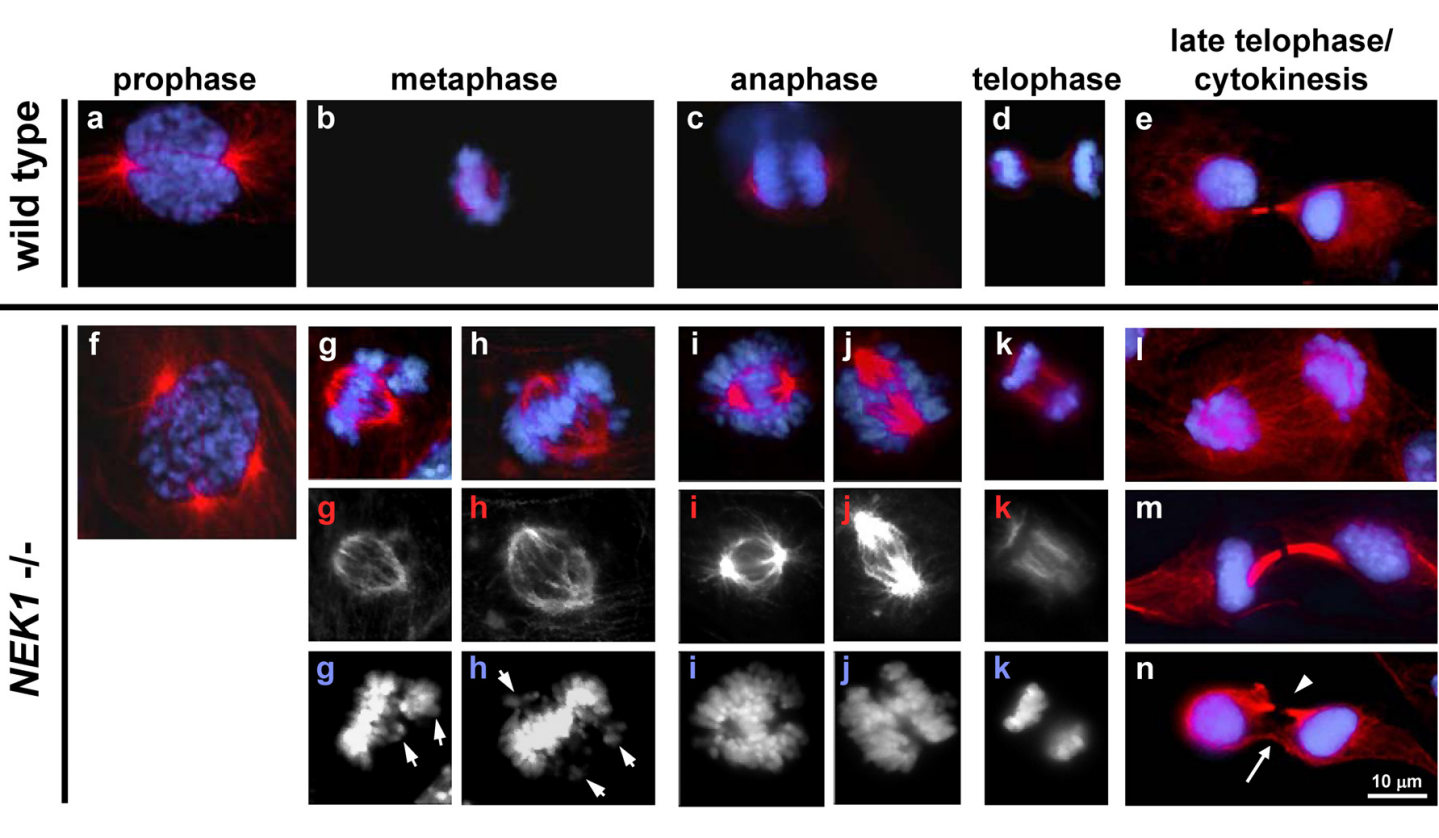
***NEK1 *mutation results in disordered mitosis**. Normal mitotic phases in primary passage 4 RTE cells from wild type littermate are shown in upper panels (a-e). DNA stained with DAPI fluoresces blue and secondarily recognized α-tubulin immunofluoresces red. In contrast, *NEK1 *-/- cells often undergo faulty and disordered mitosis (f-n), characterized by multipolar spindles (panel f), lagging chromosomes (panel g & h), improper directional movement of chromosomes or chromosome pieces during anaphase and telophase (panels i, j & k), and bizarre, incomplete types of cytokinesis (panel l, m & n). Grayscale panels are high-contrast images of the same panels above them, demonstrating individual staining for either α-tubulin or DAPI.

The aberrant nuclear morphology observed in interphase NEK1 mutant cells is a hallmark of defective mitosis. A significant population of NEK1 localizes to centrosomes, where it is thought to facilitate functions in centrosome duplication and stability maintenance [12,21]. We therefore hypothesized that the lack of NEK1 might impact centrosome function, thereby affect mitotic spindle formation, and lead to aberrant chromosome segregation. To test these possibilities, we examined the mitotic spindles and chromosomes of wild type and *NEK1 *-/- mutant cells. We found that early passage cells *NEK1 *-/- cells (passages 3 and 4) often have multipolar spindles (Figure 2f) and lagging chromosomes (Figure 2g, h), which result in improper chromosome movement (Figure 2i-k) and bizarre, incomplete cytokinesis (Figure 2l-n). These aberrant events were rarely observed in wild type cells. The aberrant mitoses in NEK1-deficient cells seemed to explain the abnormal nuclear morphology observed in many *NEK1 *-/- interphase nuclei (Figure 1).

### Aneuploidy in *NEK1 *-/- cells

**Figure 3:**
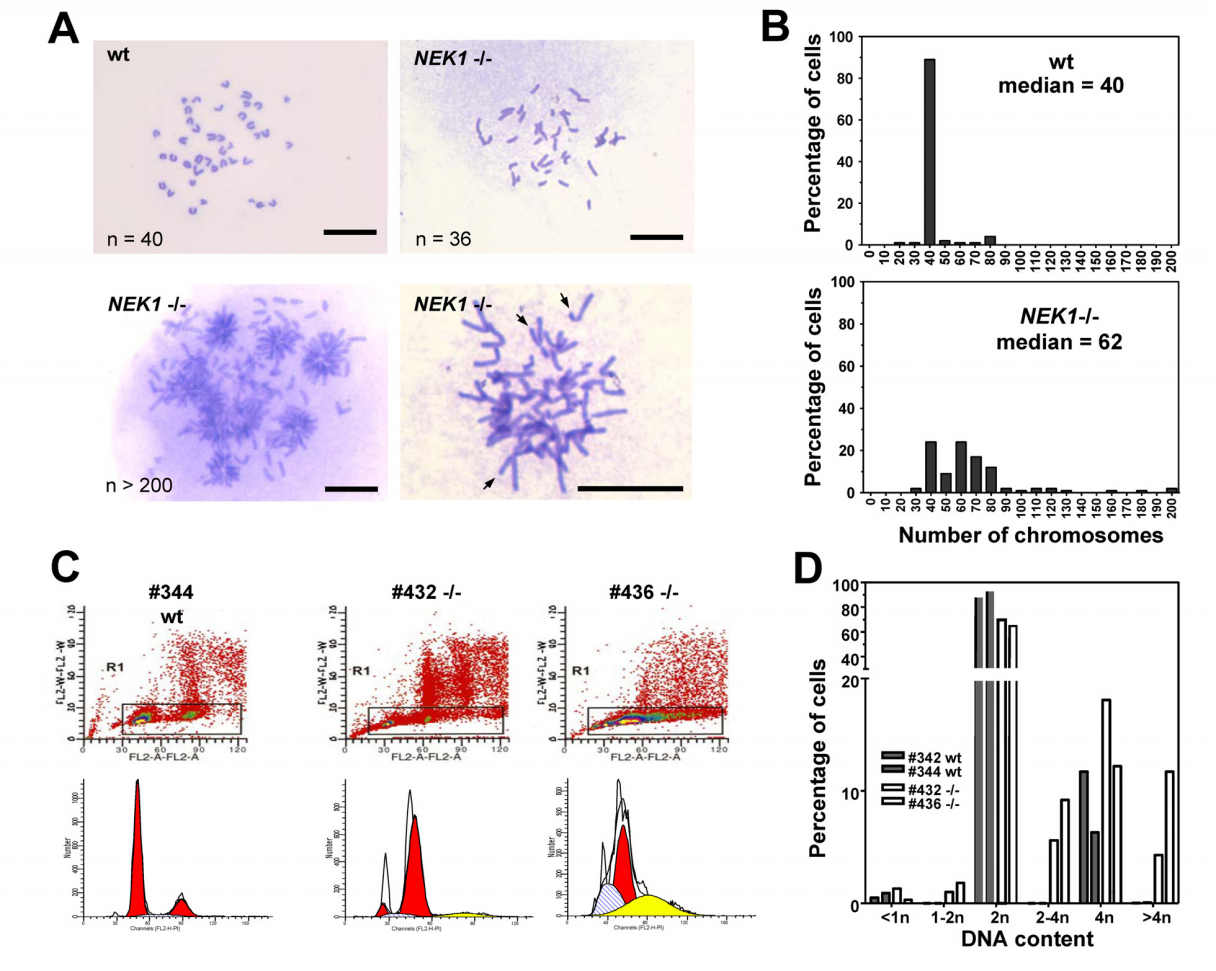
**Aneuploidy in *NEK1 *-/- cells**. (A, B). Passage 4 RTEs cultured from *NEK1 *-/- mice and from wild type littermates were treated with colchicine and hypotonically lysed. Free chromosomes were dispersed and stained with Giemsa. A. Representative images of chromosome spreads. Note the more variable sister chromatids observed in the spread prepared from *NEK1 -/- *cells. Bar = 50 μM. B. Numbers of chromosomes per cell were quantified (more than 200 spreads from at least two unique cell lines for each genotype). (C, D) Wild type or *NEK1 *-/- RTEs at passage 4 were harvested during exponential phase growth, stained with propidium iodide, and quantified by FACS for DNA content. C. Plots for wild type cells show sharp 2n and 4n peaks. One *NEK1 *-/- line (#432) shows a substantial fraction of cells with >4n DNA content (yellow). In another *NEK1 *-/- line (#436), the majority of the cells have >2n DNA content, and a broad peak is seen, representing a range of different DNA content in individual cells. D. Histograms showing 5-13% of cells with >4n DNA content and 6-9% with DNA content intermediate between 2n and 4n, specifically in *NEK1 *-/- RTEs.

We have previously shown that *NEK1 *-/- cells suffer from numerous chromosome breaks, and that NEK1 has an important role in DNA damage checkpoint control [14]. The array of abnormalities we observed in mitotic *NEK1 *-/- cells, especially the lagging chromosomes and bizarre incomplete cytokinesis, suggested a potential role for NEK1 in the spindle checkpoint. To explore the extent of chromosomal abnormalities further, we analyzed chromosome spreads. In wild type cells, 40 normal mitotic chromosomes were observed in >90% of the spreads. In contrast, only 25% of the mitotic spreads from *NEK1 *-/- cells contained 40 chromosomes (Figure 3A, B). While the vast majority of wild type cells had diploid (2n) chromosome copy number, *NEK1 *-/- cells had variable numbers of chromosomes (median = 62) (Figure 3B). Hypoploid spreads were relatively rare, especially in late passage cells, but a significant fraction (74%) of *NEK1 *-/- cells had chromosome numbers greater than 4n. These findings suggest that *NEK1 *-/- cells fail to undergo cytokinesis properly. Giemsa staining showed that chromosomes from *NEK1 *-/- cells were more variable than wild type chromosomes, and that sister chromatids (Figure 3A, arrows) were often unequal in length. This latter finding suggests chromosome rearrangements.

To analyze aneuploidy further in *NEK1 *-/- cells, fluorescence-activated cell sorting (FACS) was used to quantify the DNA content in wild type and *NEK1 *-/- cells. To control for any underlying genomic instability associated with inbreeding in the parental kat2J mouse strain, we always used untransformed cells from sex-matched, wild type littermate mice as controls. *NEK1 *-/- cells became polyploid after only a few passages, whereas wild type cells maintained consistent DNA content through dozens of population doublings in culture, until very late passages (Figure 3C, D). Fewer numbers of cells were observed to be hypoploid, as expected, since such cells would be unlikely to propagate to future generations [22].

### Transforming phenotype in *NEK1 *-/- cells

**Figure 4:**
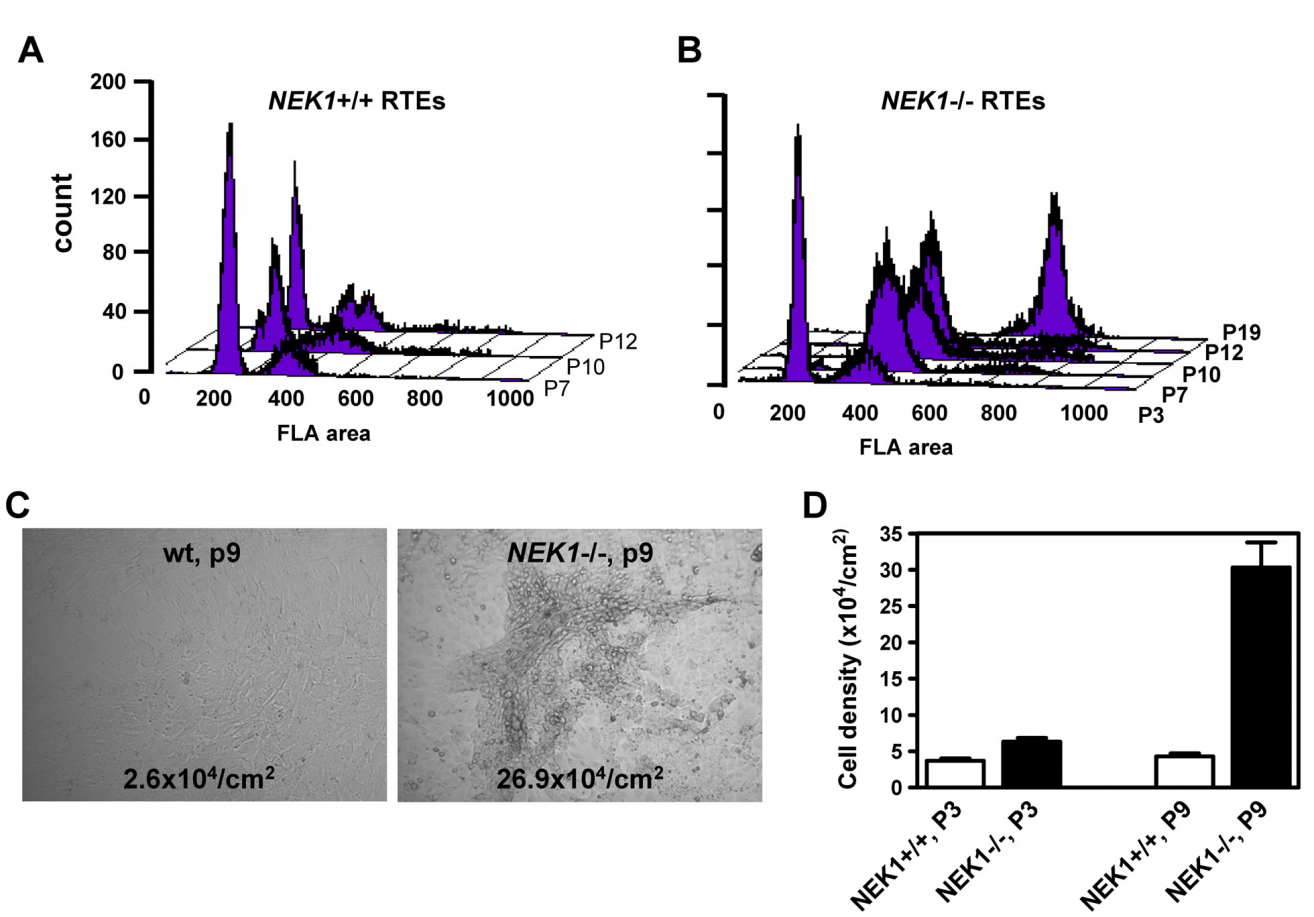
**Polyploid DNA content by FACS and lack of contact inhibition in *NEK1 *-/- cells**. RTEs cultured from *NEK1 *-/- mice and from wild type littermates were harvested at different passages during exponential phase growth, stained with propidium iodide, and quantified with FACS for DNA content. A. Wild type cells show sharp 2n and 4n peaks in early and late passage cells. B. *NEK1 *-/- cells show 2n and 4n peaks at early passages. As cells proliferate in culture, 2n population cells disappear and cells with DNA content between 2n and 4n increase in later generations. *NEK1 *-/- cells surviving at higher passages have >6n DNA content; few 2n or 4n cells are seen. (C, D.) Growth properties of wild type and *NEK1 *-/- cells. Cells at passage 3 or 9 were cultured until they reached 100% confluence, and then cultured for additional of 2 weeks, until no more proliferation was observed in wild type cells. *NEK1 *-/- cells continued to pile, beyond monolayers. C. Representative morphology of the cells immediately before harvest. D. Saturation density of cells was quantified as number of cells per cm^2^. Each histogram represents mean ± standard error from 2 different cell lines in duplicate plates.

To confirm our findings, we used FACS to analyze renal tubular epithelial cells (Figure 4) and tail fibroblasts (data not shown) derived originally from littermate mice, but passed different numbers of times in culture. In all cell types, only small numbers of wild type cells had DNA content >4n, especially in early passages. In higher passages, wild type cells still contained normal 2n and 4n populations and only small populations of cells had DNA content >4n (Figure 4A). In contrast, *NEK1 *-/- cells cultured in identical conditions and undergoing the same number of passages (and presumably the same or similar number of cell divisions), were mostly polyploid after the 4th passage (Figure 4B). Wild type cells became senescent and more difficult to propagate beyond the 12th passage, but *NEK1 *-/- cells continued to proliferate easily, suggesting that *NEK1 *-/- cells escaped senescence and became spontaneously immortalized. *NEK1 *-/- cells also exhibited other qualities of cancer cells, including loss of contact inhibition to allow for multilayered growth, and the ability to grow at higher saturation density than wild type cells (Figure 4C, D). Taken together, the many chromosomal abnormalities observed in *NEK1 *-/- cells reveal the importance of NEK1 in DNA damage checkpoints, especially in control of mitotic checkpoints. Failure to activate DNA damage checkpoints in *NEK1 *-/- cells apparently leads to chromosome instability and aneuploidy, hallmarks of neoplastic cell growth.

### *NEK1 *-/- cells grow without anchorage dependence and form tumors when injected into syngeneic mice

**Figure 5:**
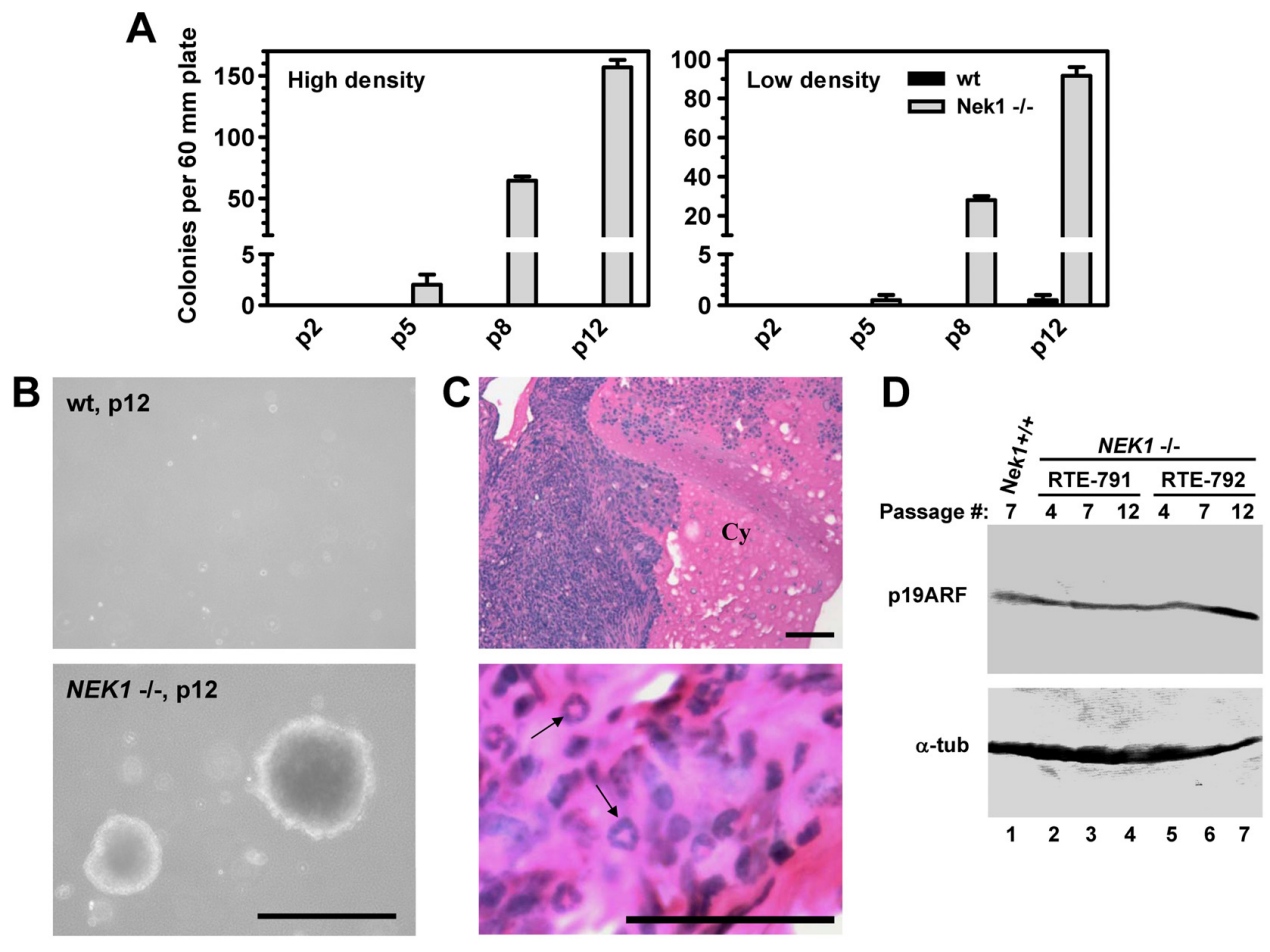
**Cultured *NEK1 *-/- cells form colonies in soft agar and tumors in when injected into mice**. A, B. Equal numbers of unclumped cells (high density (1 × 10^5^/60 mm); low density, (2 × 10^4^/60 mm) of the indicated passage were seeded in duplicate in 0.367% agar. Total colony numbers were scored after 21 days. Contiguous clusters of >50 cells were considered colonies. Representative colonies were photographed as shown in B. Bar = 100 μm. C. H&E stained sections of tumors, 21 days after subcutaneous injection of *NEK1 *-/- RTEs into kat2J mice. Cy = cyst, arrows = abnormal nuclei. Bars = 100 μm. D. p19ARF expression in two distinct *NEK1 *-/- cell lines at different passages in culture, and in wild type (*NEK1 *+/+) cells at passage 7. Alpha tubulin expression served to control for protein loading in each lane.

The phenotypes we observed in *NEK1 *-/- cells prompted us to look for neoplastic behavior. A good predictor of whether cancer cells can form tumors in vivo is their ability to grow in soft agar without attachment to plastic. We therefore employed the soft agar assay to look for anchorage-independent colony formation in *NEK1 *-/- and wild type RTEs derived from sex-matched, littermate mice and cultured in identical conditions. At different passages, well-separated cells were seeded into soft agar and examined for their ability to form colonies. As early as passage five, small numbers of *NEK1 *-/- cells were observed to form colonies. At higher passages, *NEK1 *-/- cells were even more efficient in forming colonies. In contrast, wild type cells grew poorly and failed to proliferate beyond the single cell stage in the soft agar, even after many passages in monolayer culture (Figure 5A). NEK1 inactivation therefore seems to lead to a transformed phenotype in clones of cultured cells.

To extend our observation, we injected *NEK1 *-/- or wild type RTEs into kat2J mice and analyzed the cells' ability to form tumors in vivo. Since *NEK1 *-/- cells at passage 7 already were polyploid by FACS analysis, and since they were able to form colonies in soft agar, cells at this passage were used. Seven days after injection, *NEK1 *-/- cells formed visible tumors at injection sites. Wild type cells did not form any palpable tumors. At day 10 after injection, the tumors reached maximum size, and thereafter shrank slightly (mean tumor volume was 193 μl at day 10 (n = 4) and 137 μl (n = 6) at day 21). Tumors were harvested and examined histologically. Cysts were observed in the tumors grossly and confirmed by microscopic examination (Figure 5B). There was no histological evidence of unusual neo-vascularization, nor any major changes in vascularization between day 10 and day 21 tumors. Many of the cells within the somewhat pleomorphic tumors had bizarre, ring nuclei (Figure 5C) reminiscent of the hollow nuclei seen in cultured *NEK1 *-/- RTEs. The histological features of the tumors were similar to those observed in colonies grown in soft agar (not shown). To help determine whether the p16INK/p19ARF pathway, which is commonly affected in cancers [23], is downregulated in tumors mediated by Nek1 deficiency or inactivation, we examined p19ARF in *NEK1 *-/- cells. p19ARF expression was not altered (Figure 5D) in *NEK1 *-/- cells, even after multiple passages. This result suggests that NEK1 inactivation leads to tumor formation independent of p19ARF.

### Spontaneous tumors in *NEK1 *+/- mice

**Figure 6:**
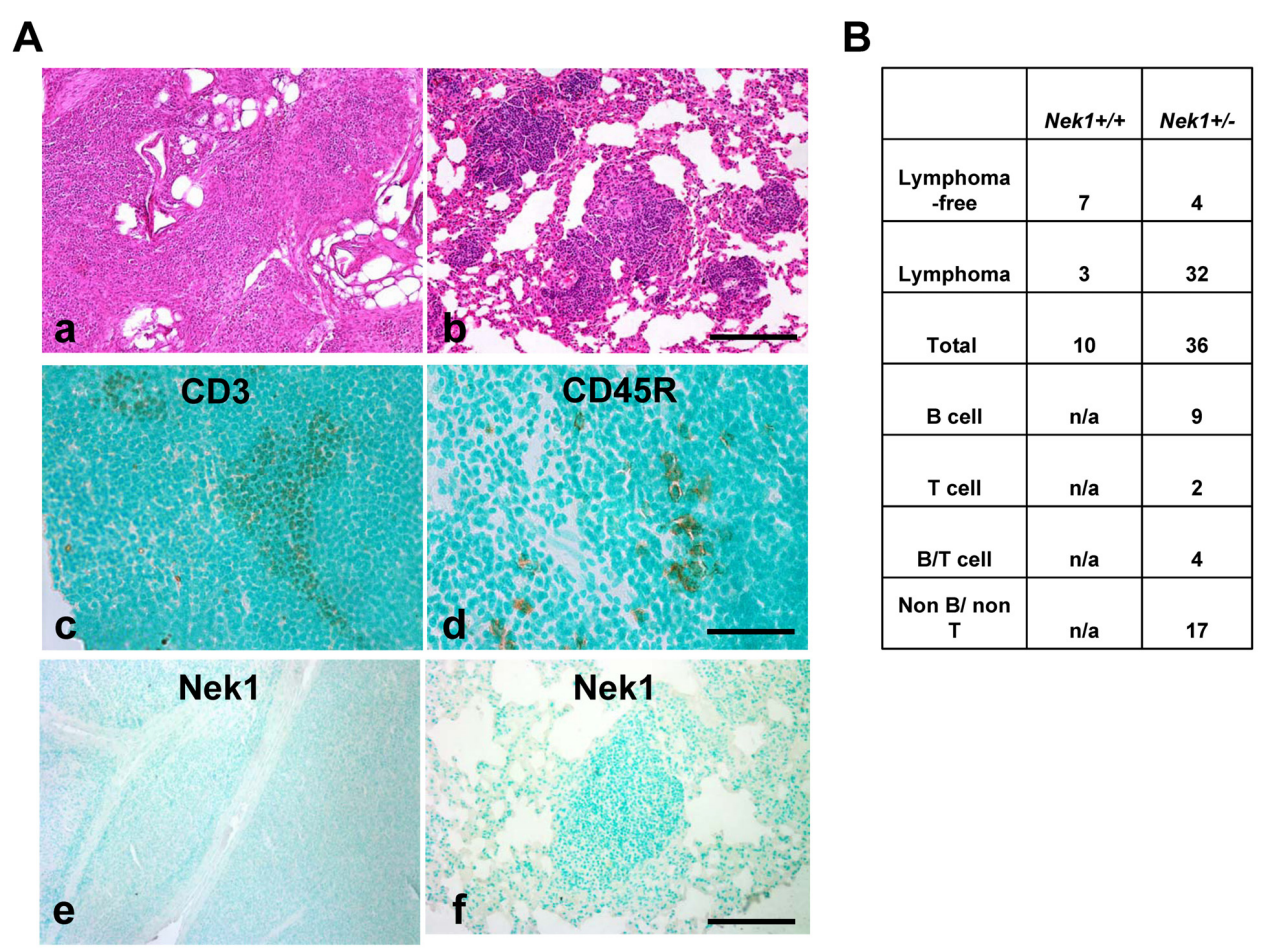
**Spontaneous lymphoid tumors in *NEK1 *+/- mice**. *NEK1 *+/- and wild type mice were sacrificed at ages 17-24 months. Lymphoid tumors were observed much more frequently in *NEK1 *+/- mice than in wild type littermates. A. Representative H&E stained sections of lymphoid tumors, one from a mesenteric lymph node (panel a) and one within lung parenchyma (panel b). The same tumors were stained with anti-CD3 to mark T-lymphocytes (panel c) and anti-CD45R to mark B-lymphocytes (panel d). Representative sections of lymphoid tumors stained in with rabbit anti-NEK1 antibody: lymphoid tumor from a mesenteric node (panel e) and from a lung (panel f). Bar = 100 μM (panels a, b, e, f); 50 μM (panels c, d). B. Summary table of lymphoid tumors found in aged *NEK1 *+/- *kat2J *mice.

NEK1 deficient cells segregate their chromosomes aberrantly and gain a polyploid, transformed phenotype. In vivo, *NEK1 *-/- kat2J mice develop polycystic kidney disease at a young age and often do not survive past 3 weeks after birth (unpublished result). In the few *NEK1 *-/- kat2J mice that we did observe to survive to two months of age, 50% (3/6) developed lymphomas and the other 50% (3/6) had lymphoid aggregates in various organs. Since we have shown that *NEK1 *+/- cells are more sensitive to IR than cells from wild type littermates [15], we know that haploid insufficiency for *NEK1 *also impairs DNA damage response and repair, even if it does so to a lesser degree than complete inactivation of NEK1 in null mice and cells. We hypothesized therefore that *NEK1 *+/- kat2J mice should be prone to develop cancers spontaneously. To examine this possibility, we monitored tumor development in a cohort of *NEK1 *+/- kat2J mice and wild type mice as they aged. By 17-24 months of age, 30% of wild type mice (3/10) and 89% of *NEK1 *+/- kat2J littermate mice (33/36) developed lymphoid tumors. Tumors were discovered in many lymphatic chains, but most prominently in mesenteric nodes. Some of the tumors invaded or metastasized to parenchymal tissues such as lungs (Figure 6A). This lymphoid tumor phenotype demonstrates, in vivo, that haplo-insufficiency for *NEK1 *leads spontaneously to cancer. We characterized the lymphoid tumors or lymphomas in *NEK1 *+/- mice by immunohistochemical analysis, using anti-CD3 antibodies for T-cells and anti-CD45R antibodies for B-cells. The majority of the tumors (17 of 32) failed to stain for either CD3 or CD45R. Nine of 32 stained positively for CD45R (B-cells), 2 were positive for CD3 (T cells), and 4 were positive for both CD3 and CD45R (B- and T-cells) (Figure 6A, B). Most cells in lymphoid tumors from *NEK1 *+/- kat2J mice stained negatively for NEK1 (Figure 6A, panels e, f). This lack of staining suggests that immunologically recognizable NEK1 may be lost in somatic cells that acquire the transformed phenotype.

**Figure 7:**
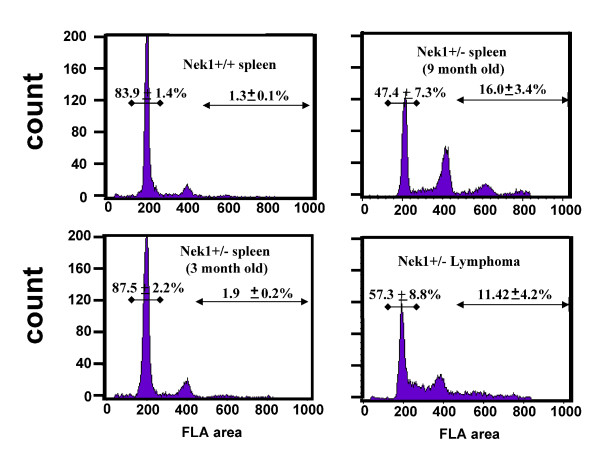
**Anueploidy in lymphoid tumors from older *NEK1 *+/- mice**. Cells from the spleens of a 9 month-old wild type mouse and a *NEK1 *+/- mice littermate, 3 month-old *NEK1 *+/- mice, and three lymphoid tumors were fixed and stained with propidium iodide, and then analyzed by FACS for DNA content. Percentages of 2n cells from the spleen from old *NEK1 *+/- mice and from a lymphoma were decreased compared to cells from the spleens of wild type and young *NEK1 *+/- mice. Cells with DNA content greater than 4n were increased in the spleen cells from old *NEK1 *+/- mice and in the lymphoid tumors. In the lymphoid tumor shown, note also the relatively large number of cells, characterized by high valleys in the FACS plots, with non-integer DNA content. See also additional file 1-Fig. S1.

Analysis of a limited number of lymphoid tumors from *NEK1 *+/- mice by FACS revealed that the cells comprising them, and even some splenic lymphocytes in older *NEK1 *+/- mice, have aberrant DNA content (Figure 7 and additional file 1-Fig. S1). Cells from wild type spleens and from spleens of young *NEK1 *+/- mice without visible tumors served as controls. The cells from the spleens of older mice had more than usual complements of cells with 6n and 8n DNA content. Cells from lymphoid tumors had not only polyploid cells with 6n and 8n DNA, but many cells with non-integer DNA content, characteristic of chromosome pieces. In this regard, the lymphoid tumors from *NEK1 *+/- mice were similar to the *NEK1 *-/- cells in culture (compare with Figure 3C).

## Discussion

Our earlier studies have suggested a role for NEK1 in early DNA damage response and cell cycle checkpoint activation [14,15]. The data presented here demonstrate that cells deficient in NEK1 develop chromosomal abnormalities during mitosis, and become aneuploid after several division cycles. These abnormal cells gain the ability to overcome density-dependent growth inhibition, to grow in anchorage independent conditions, and to form tumors in mice, i.e., they become malignant. The studies in cultured cells were designed to represent an accelerated version, telescoped in time, of what happens to NEK1-deficient cells in vivo as they're exposed to oxidative stresses and other DNA-damaging injuries. Interestingly, lymphoid tumor cells from older *NEK1 *+/- mice had distinct, polyploid DNA contents very similar to those seen in *NEK1 *-/- cells passed several times in culture. Experiments using a complementary technique, FACS analysis in human U2OS tumor cells with stable knock-down of NEK1 expression, showed that NEK1 deficiency, not only the specific *NEK1/kat2J *mutation, leads directly to acquisition of the polyploid phenotype (additional file 2 Fig. S2B).

In kat2J mice with only one normal allele of *NEK1*, we observed an important consequence of defective DNA damage repair and chromosome instability: a very high incidence of tumors, in particular lymphomas. The cumulative incidence of lymphomas in inbred C57Bl/6 mice is reported to be 30% [24]. We observed the same incidence in our wild type mice, but a 3-fold greater incidence in identically maintained *NEK1 *+/- littermates. The lymphomas were heterogeneous, deriving from B-, T-, and unclassifiable lymphocytes, suggesting defects in repair of damaged DNA and in mitotic segregation in multiple lineages and maturation steps, not in any one particular lymphocyte differentiation pathway. *NEK1 *null mice also developed lymphomas, but most of them died at early ages from other causes. Lymphomas or lymphoproferative disorders are common tumors in mammals with DNA damage sensing or repair defects [25-31]. Clones of lymphoid cells have to divide multiple times, and they undergo frequent gene rearrangements as they respond to different antigen epitopes throughout the life of an animal. Many lymphocytes should also undergo apoptosis as part of normal process of clonal deletion. Lymphoid tumors therefore are especially prone to acquire mutations, and to proliferate and become malignant if they escape normal cell cycle checkpoints, DNA damage repair mechanisms, or pathways to appropriate programmed death.

The data we present here and in previous reports [14,15] concerning NEK1's role in DNA damage and mitotic checkpoint control is similar to what is known about fungal NIMA. *Aspergillus Nima *mutants never enter mitosis. Instead, they arrest in late G2 phase with duplicated spindle pole bodies, the fungal equivalents of mammalian cell centrosomes [32]. Overexpression of NIMA in *Aspergillus*, yeast, and even in human cells, causes premature condensation of chromosomes independent of CDC2, the proximal cyclin dependent kinase controlling access to M phase [2,33-35]. If *nimA5 Aspergillus *mutants are forced to bypass G2/M phase checkpoint arrest by acquisition of an additional mutation in bimE7, an anaphase-promoting complex subunit APC1 [36], they develop grossly abnormal mitotic phenotypes: multiple spindle pole bodies, spindles with disparate sizes and shapes, disorganized microtubules arranged in multiple directions other than orthogonal ones, and defects in nuclear envelope structure and in nucleokinesis [4]. We observed similarly aberrant mitoses in murine *NEK1 *-/- cells after only a few division cycles in culture. Our observations are intriguing, for in mammalian cells NEK1 deficiency alone results in mitotic segregation errors and aneuploidy, whereas bypassing the *nimA5 *mutation in *Aspergillus *requires an additional mutation in *bimE7 *to get the cells past a mitotic checkpoint.

Our data suggests that NEK1 is not essential for entry into mitosis, but instead that it is important for regulating the timing and fidelity of chromosome segregation via its role in centrosomes and in generating bipolar, orthogonal, mitotic spindles. Our studies highlight the multifaceted nature of NEK1 function in DNA damage checkpoint control and centrosomal function. Whether the phenotypes observed in *NEK1 *-/- cells are due to centrosome maintenance directly, to checkpoint activation, or both will require further investigation. NEK1 does localize to centrosomes, where it is thought to affect the stability of primary cilia and centrosomes directly, as well as cell cycle progression indirectly through signaling and transcriptional programs [12,21]. Different regions within NEK1's kinase and coiled-coil domains have been shown using overexpressed, GFP-tagged, deletion constructs to affect localization of NEK1 to primary cilia and to γ-tubulin in centrosomes [12,21]. These experiments were performed in murine renal inner medullary collecting duct (IMCD3) cells, which express normal amounts of endogenous NEK1, so they were not be able to address differential effects of the various NEK1 deletion mutants on chromosomal maintenance. To address this issue in light of our findings of chromosome instability in NEK1-deficient cells, identification and refinement of mutants that differentially affect only NEK1 in centrosome function, in checkpoint activation, or in the DNA damage repair pathway will need to be made. These mutants would then need to be added back to *NEK1 *-/- cells in order to dissect the specific, differential functions of NEK1 in chromosomal and checkpoint control phenotypes. We have started such experiments.

Cells that divide without functional NEK1 develop major defects in mitotic spindle function, thus compromising the cells' ability to segregate chromosomes faithfully to the two daughter cells, and resulting in chromosome rearrangements and aneuploidy [37,38]. *NEK1 *-/- cells frequently have aberrant mitotic spindles with lagging and misaligned chromosomes (Figure 2). Such gross chromosomal rearrangements would result in aneuploid cells that acquire copies of oncogenes or lose tumor suppressors. Only a small subset of these cells would gain a growth advantage and continue to proliferate, whereas the majority of such cells, especially those with too few chromosomes, would die by apoptosis. Polyploid cells gaining growth advantages eventually would become the survivors, and they would proliferate without proper regulation, as malignant cells do. Polyploid *NEK1 *-/- cells are particularly remarkable in that they do not always have integer multiples of n chromosomes. Instead, they often have pieces of chromosomes and DNA content between 2n and 4n, or between 4n and 6n (Figure 3), as well as micronuclei, multiple nuclei of different sizes, or hollow nuclei in interphase cells (Figure 1). These unique phenotypes suggest that NEK1 deficiency affects not only cytokinesis, but also affects sister chromatid pairing and chromosomal rearrangements like translocations, losses, and losses with reduplications.

An argument can be made that the aneuploid phenotype observed in *NEK1 *-/- cells could be a secondary effect, due to additional mutation in another locus. Addressing this issue has been challenging, since re-expression of wild type NEK1 in *NEK1 *-/- cells induces checkpoint activation, such that those cells don't continue to proliferate (additional file 2-Fig. S2A) and such that mitotic cells are not observed. Differentiating direct from secondary events might require in vivo experiments, with transgenic mice overexpressing NEK1 crossed into the *NEK1/kat2J *-/- background, where the mice could be observed for rescue of tumor formation and genomic instability phenotypes.

A growing body of evidence from mouse models has linked abnormal DNA damage repair and disturbed mitotic events to the genesis and progression of cancer (for review see [39]). NEK1 does not appear to be absolutely required for development in mice, which seem not to acquire prominent aneuploid phenotypes during embryonic stages. Excessive apoptosis is evident in the kidneys and other organs of embryonic and newborn *NEK1/kat2J *-/- mice, however (manuscript in preparation). This observation suggests that cells with abnormal chromosomes content are eliminated during development. NEK1's role in DNA damage responses may be subtle and regulatory, akin to the role associated with the key DNA damage response kinase ataxia telangiectasia mutated (ATM) [40]. Nek1-null mice seem to be in important ways similar to Atm-deficient mice and humans with ataxia-telangiectasia, which survive embryonic and early adult stages, but which age prematurely and develop lymphomas and other tumors later in life as they're exposed to environmental insults [26]. The ATM and Rad3-related kinase (ATR), in contrast to ATM and NEK1, is more fundamental in signaling pathways required for recognition and repair of DNA replication intermediates, and results in early embryonic lethality with fragmented chromosomes when mutated [41]. We suggest that repeated injuries over time may need to accumulate in order to manifest gross chromosomal abnormalities and cancers late in the life of a NEK1-deficient animal, as in the life of an ATM-deficient animal. Such injuries would not occur much during embryonic development.

Our current results showing that mice heterozygous for *NEK1 *have a high cumulative incidence of lymphomas, derived from all types of lymphocytes, suggests that low level expression of NEK1 in cells expressing it from a single allele is not sufficient to safeguard the genome and prevent chromosome instability. Since NEK1 is important for DNA damage response/repair and centrosome maintenance, the expression of sufficient amounts of NEK1 might be required for proper mitotic checkpoint activation and for assuring precise mitotic chromosomal segregation and cellular cytokinesis. Studying the level of NEK1 expression in different human cancers will help to determine whether chromosome instability observed in these cancers can be attributed to loss of NEK1 activity, and whether NEK1 could be an important target for cancer treatment. We know of no published studies to date that have implicated NEK1 mutations in the pathogenesis of human tumors. Further studies on whether diminished NEK1 expression leads to tumor formation in humans should be explored.

## Conclusions

NEK1 is required for maintaining chromosome stability. Cells without functional NEK1 generate abnormal mitotic spindles with lagging chromosomes, become aneuploid, overcome density-dependent growth inhibition, proliferate without anchorage dependence, and gain the ability to form tumors when injected into syngeneic mice. NEK1 deficiency and the chromosome instability associated with it also have consequences in vivo: *NEK1 *heterozygous mice develop lymphomas with a much higher incidence than wild type littermates. These observations, when combined with our previous reports about the role of NEK1 in DNA damage checkpoint control, suggest that NEK1 is important for safeguarding the genome, by participating in the DNA damage and responses, and by assuring faithful chromosome segregation during mitosis.

## List of abbreviations

NIMA: never in mitosis mutant A; NEK1: NIMA-related protein kinase 1; RTE: renal tubular epithelial cell; IR: ionizing radiation; CIN: chromosome instability; DAPI: 4',6-diamidino-2-phenylindole; SSCP: single-strand conformational polymorphism; UBC: ubiquitin C

## Competing interests

The authors declare that they have no competing interests.

## Authors' contributions

YC conceived, designed and coordinated the study, acquired, analyzed, and interpreted data, and helped draft the manuscript. CFC performed and acquired the FACS analysis data. HCC and MP performed or helped interpret the histological sections. RP performed the experiments in cultured cells. RLW helped draft the manuscript and participated in discussions. RAE and DEH participated in interpretation of the analysis of histological sections. PLC participated in the interpretation the data, discussion, and drafting of the manuscript. DJR conceived, designed, acquired, analyzed, and interpreted data, and drafted the manuscript. All authors read and approved the final manuscript.

## Supplementary Material

Additional file 1 **Figure S1: Anueploidy in lymphoid tumors from older *NEK1 *+/- mice**. FASC analysis results of spleens and lymphoid tumor. Raw height and FL2 area plots of cells from representative spleens and a lymphoid tumor. Note the scatter in the lymphoid tumor cells, representing cells with non-integer DNA content. Click here for file

Additional file 2 **Figure S2: Cell cycle arrest of NEK1 -/- cells after retroviral-mediated expression of wild type NEK1 and aneuploidy in NEK1 knocked down cells**. Re-expression of NEK1 into NEK1-/- cells induced cell cycle arrest and silencing NEK1 expression in U2-OS cells increases polyploidy in higher passage cells.A. FACS analysis of NEK1 -/- cells after retroviral-mediated expression of wild type NEK1. Nek1/kat2J -/- cells were infected with a retrovirus expression vector for NEK1 under control of a UBC promoter. The expression of NEK1 was detected by Western blotting. Expression of p84 served as a control for loading. Five days after infection, cells were fixed, immunostained with anti-phospho-H3 antibodies, and anaylyzed by FACS. B. U2-OS cells with NEK1 expression knocked down by stable RNA silencing. U2-OS cells were transfected with a NEK1 shRNAi construct. Stable NEK1 knockdown cells, N27, were selected and propagated. At passages 5 and 7, the cells were fixed and analyzed by FACS. Increasing polyploidy was evident in the higher passage N27 cells. Click here for file
