# Supplementary figures and images for: Mutation of NIMA-related kinase 1 (NEK1) leads to chromosome instability
Source: Mol Cancer. 2011 Jan 10;10:5. doi: 10.1186/1476-4598-10-5 (PMC3025975; doi:10.1186/1476-4598-10-5)

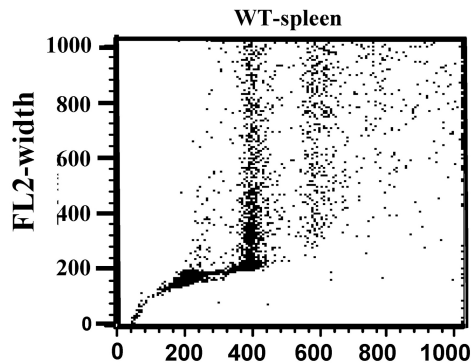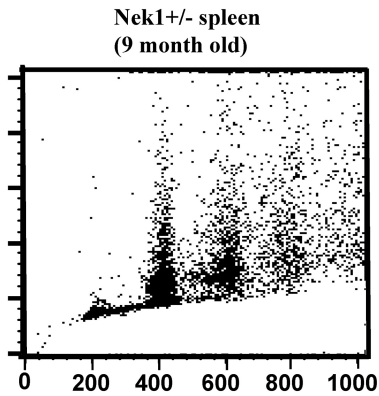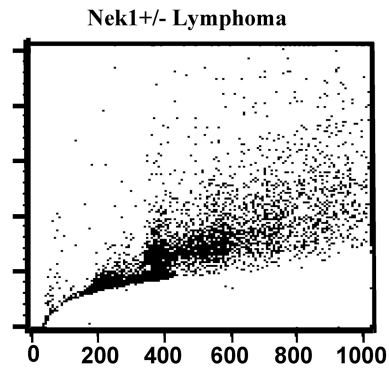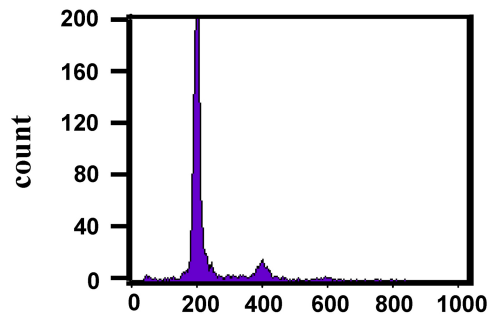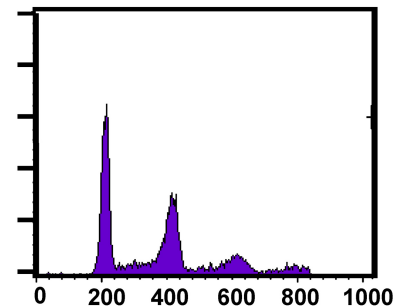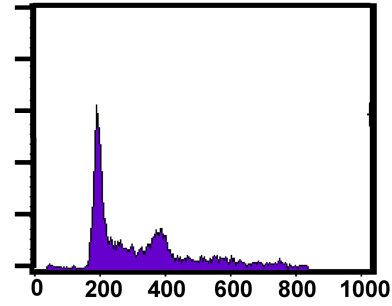

**FL2-area**

Supplement: Additional file 1 — Figure S1: Anueploidy in lymphoid tumors from older NEK1 +/- mice. FASC analysis results of spleens and lymphoid tumor. Raw height and FL2 area plots of cells from representative spleens and a lymphoid tumor. Note the scatter in the lymphoid tumor cells, representing cells with non-integer DNA content. [file 1476-4598-10-5-S1.PDF]

**A**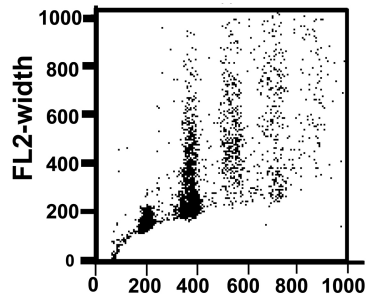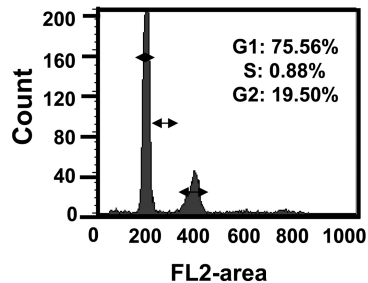**B**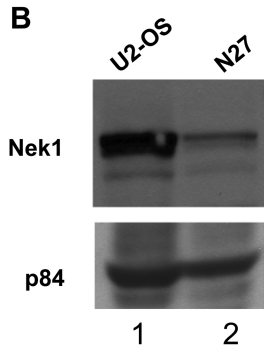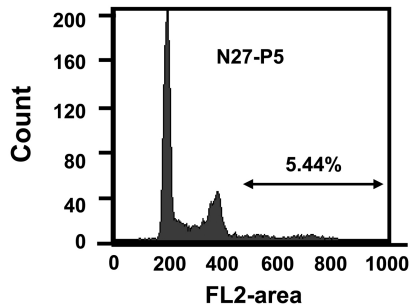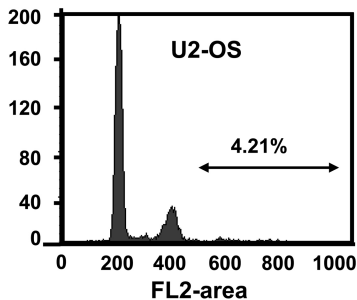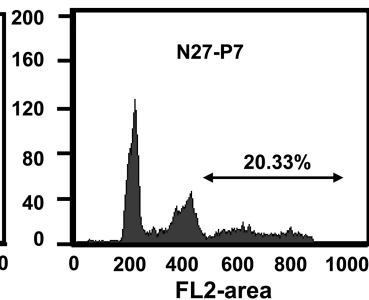

Supplement: Additional file 2 — Figure S2: Cell cycle arrest of NEK1 -/- cells after retroviral-mediated expression of wild type NEK1 and aneuploidy in NEK1 knocked down cells. Re-expression of NEK1 into NEK1-/- cells induced cell cycle arrest and silencing NEK1 expression in U2-OS cells increases polyploidy in higher passage cells.A. FACS analysis of NEK1 -/- cells after retroviral-mediated expression of wild type NEK1. Nek1/kat2J -/- cells were infected with a retrovirus expression vector for NEK1 under control of a UBC promoter. The expression of NEK1 was detected by Western blotting. Expression of p84 served as a control for loading. Five days after infection, cells were fixed, immunostained with anti-phospho-H3 antibodies, and anaylyzed by FACS. B. U2-OS cells with NEK1 expression knocked down by stable RNA silencing. U2-OS cells were transfected with a NEK1 shRNAi construct. Stable NEK1 knockdown cells, N27, were selected and propagated. At passages 5 and 7, the cells were fixed and analyzed by FACS. Increasing polyploidy was evident in the higher passage N27 cells. [file 1476-4598-10-5-S2.PDF]
